# Typhoid Control in an Era of Antimicrobial Resistance: Challenges and Opportunities

**DOI:** 10.1093/ofid/ofad135

**Published:** 2023-06-02

**Authors:** Samantha Vanderslott, Supriya Kumar, Yaw Adu-Sarkodie, Firdausi Qadri, Raphaël M Zellweger

**Affiliations:** Oxford Vaccine Group, Department of Paediatrics, University of Oxford, Oxford, United Kingdom and NIHR Oxford Biomedical Research Centre, Oxford, Oxfordshire, United Kingdom; Enteric and Diarrheal Diseases, Bill & Melinda Gates Foundation, Seattle, Washington, USA; Kwame Nkrumah University of Science and Technology, Kumasi, Ghana; International Centre for Diarrhoeal Disease Research, Dhaka, Bangladesh; Epidemiology, Public Health and Impact, International Vaccine Institute, Seoul, South Korea

**Keywords:** salmonella, typhoid, typhoid conjugate vaccines, typhoid control

## Abstract

Historically, typhoid control has been achieved with water and sanitation interventions. Today, in an era of rising antimicrobial resistance (AMR), two World Health Organization-prequalified vaccines are available to accelerate control in the shorter term. Meanwhile, water and sanitation interventions could be implemented in the longer term to sustainably prevent typhoid in low- and middle-income countries. This article first approaches typhoid control from a historical perspective, subsequently presents how vaccination could complement water and sanitation activities, and finally discusses the challenges and opportunities for impactful control of typhoid infection. It also addresses data blind spots and knowledge gaps to focus on for typhoid control and to ultimately progress towards elimination. This article presents a synthesis of discussions held in December 2021 during a roundtable session at the “12th International Conference on Typhoid and Other Invasive Salmonelloses”.

## Teaser

This article discusses challenges and opportunities related to typhoid control and elimination in an era of rising drug resistance. We propose that vaccines are an invaluable tool to complement other approaches such as investment in water, sanitation, and diagnostic improvement.

## HISTORICAL PERSPECTIVE ON TYPHOID CONTROL

The elimination of typhoid fever has depended in part on new technologies to prevent, diagnose, and treat the infection. By the early 1900s, many cities in the Global North were investing in water and sanitation infrastructures. Overall, there has been a correlation [1] between rising expenditure on the provision of safe water services (including treating the water supply with chlorine) [2] and declining mortality from waterborne diseases such as typhoid. Beginning in 1896, vaccines were also developed to protect populations in areas without sanitary infrastructure. By World War I, all major powers [3] used typhoid vaccines to protect troops and travelers.

At the same time, research on typhoid showed that transmission was more complex than initially thought. Researchers discovered that the bacterium could be excreted by people who appeared to be healthy. These so-called asymptomatic—or healthy—carriers have no symptoms but can still excrete *Salmonella* Typhi through their feces for years after initial infection. The concept of healthy carriers was advanced by the German bacteriologist Robert Koch in 1902 [4] and stalled hopes for typhoid elimination because these asymptomatic and outwardly “healthy” people could be putting others at risk. Most typhoid carriers were allowed to remain in their communities if they agreed to follow precautionary hygiene measures (such as abstaining from working in food preparation and waterworks), but some were forcibly detained and isolated. Famously, the Irish immigrant Mary Mallon who became known as “Typhoid Mary” [5] was detained after repeatedly infecting those for whom she cooked.

By the end of World War II, Europe and North America had functioning sanitation systems, chlorination, fine-grained national surveillance for typhoid outbreaks and carriers by public health authorities, vaccines, and the advent of effective antibiotics [6] (chloromycetin in 1948 and ampicillin in 1961). Although typhoid has almost disappeared from high-income countries and is declining in some middle-income countries, it remains endemic in many low-resource settings [7]. This infectious divide has been reinforced by a relative neglect of international campaigns to tackle typhoid [8], such as the sustained, large-scale investment in the supply of safe drinking water, safe sewage disposal, and basic healthcare services. Investment has often remained ad hoc, uncoordinated, and insufficient, with many high-income countries focusing on protecting their own populations by prioritizing vaccines, antibiotics, and surveillance-based biosecurity regimes to stop typhoid from crossing back into high-income countries via travelers and migrants [8]. Although investment in long-term solutions has been lacking, the overreliance on comparatively cheap antibiotics to keep the disease in check has resulted in an evolutionary surge of increasingly antibiotic-resistant typhoid strains [9].

## VACCINE AS A TOOL FOR CONTROL IN THE CONTEXT OF GROWING RESISTANCE

To compound a lack of investment in long-term solutions, the control and treatment of typhoid is becoming more complex due to widespread multidrug resistance (MDR) to ampicillin, chloramphenicol and co-trimoxazole and increasing fluoroquinolone nonsusceptibility (FQNS). The intensity of this situation is magnified by the emergence and international spread of extensively drug-resistant (XDR) *S.* Typhi, which are not susceptible to at least 5 antibiotic classes, lacking sensitivity to even third-generation cephalosporins as well as ampicillin, chloramphenicol, co-trimoxazole, and fluoroquinolones. These variants have emerged and spread in Pakistan, with cases being reported in travelers globally [10–12]. The Pakistani XDR strain is proving resistant to all commonly available antibiotics except azithromycin.

In a recent study, the Surveillance for Enteric Fever in Asia Project (SEAP) has reported resistance rates for *S.* Typhi in Pakistan (16% MDR, 64% XDR, 95% FQNS), Bangladesh (17% MDR, 98% FQNS), and Nepal (1% MDR, 87% FQNS) for the period September 2016 to September 2019 [13]. Another study in a tertiary hospital in Pakistan reported that of 600 blood cultures positive for *Salmonella* from May 2020 to February 2021, 147 were MDR *S.* Typhi (24.5.%) and 276 were XDR *S.* Typhi (46.1%) [14]. A striking example of the rise of FQNS comes from Nepal, where a 23-year-long retrospective study highlighted a steep rise in ciprofloxacin nonsusceptibility in *S.* Typhi from almost none until 2009 to almost 100% in 2014 [15].

International trade and travel make it inevitable that a regional rise of antibiotic resistance will have global knock-on effects. This problem is further intensified by underreporting and international surveillance gaps, meaning that drug-resistant typhoid may be even more extensive than current estimates. It is concerning that in 2021, 9 cases of XDR-typhoid [16] were identified in the United States that were *not* linked to travel. It is likely these nontravel-related resistant cases will increase. Many would have thought a disease such as typhoid would no longer afflict higher income countries, because sanitation improvements, effective vaccines, and antibiotics had previously eliminated endemic typhoid. However, the inward-looking nature of Western disease control efforts over the last century has meant that although typhoid control stopped at high-income borders, typhoid endured as a neglected disease in other, poorer countries [8]. As research [17] shows, global neglect is now proving costly, as demonstrated by the high typhoid burden still present in many low- and middle-income countries [18], as well as the high economic impact of typhoid (both in terms of costs and loss of productivity) demonstrated by several cost of illness studies in Asia and Africa [19–23].

As a result of the alarming MDR and XDR situation (eg, close to 6500 estimated deaths attributable to MDR *S.* Typhi in 2019 [24]), the use of World Health Organization (WHO-prequalified) and effective typhoid conjugate vaccines (TCVs) appear to be one important step to limit the spread of *S.* Typhi [13, 25, 26]. Indeed, Typbar-TCV (from Bharat Biotech) requires a single dose to offer protection, as shown in a recent phase 3 trial in Nepal demonstrating 79% efficacy against blood culture-confirmed typhoid fever at 2 years [27], and in a study in Malawi reporting an efficacy of 80.4% 3 years after vaccination [28]. Data from long-term follow-up studies suggest long-lasting immunogenicity and elevated antibody titers up to 7 years after a single vaccination, with or without a booster dose [29]. More data on duration of protection and the potential need for a booster are expected in the next 1–2 years [30].

Since 2017, the Strategic Advisory Group of Experts (SAGE) from the WHO recommends the use of TCVs in endemic countries as a single routine dose for infants and children over 6 months of age (and catch-up dose for children up to 15 years when feasible), as well as after outbreaks, and for individuals at high risk of transmission [31, 32]. Two TCVs have currently obtained WHO prequalification: Typbar TCV, which uses Vi-polysaccharide conjugated to tetanus toxoid (Vi-TT) [33], and Typhibev (Biological E), consisting of Vi-polysaccharide conjugated to the diphtheria toxoid carrier CRM197 [34]. As part of its “Leaving no one behind with immunization” by 2030 strategy, Gavi [35] will continue to support TCVs for the strategic period 2021–2025 for routine, campaign, and outbreak response use. To date, several countries have introduced it, eg, Pakistan (2019), Liberia (2021), Zimbabwe (2021), and Nepal (2022) [35, 36].

One clear benefit of the current TCVs is that they are approved for use in children 6 months of age and older [30], whereas previous polysaccharide typhoid vaccines were not suitable for children under 2 years due to poor immunogenicity [34]. The impact of these vaccines is noteworthy because they protect infants, children, and adults. The spread of the resistant pathogens can be curbed by decreasing infection and/or shedding in vaccinated individuals, thereby also limiting transmission to nonvaccinated individuals via indirect effects [37]. In addition, vaccines are used preventively before infection and are usually less likely to induce resistance in the targeted pathogens compared with antimicrobials, which are typically prescribed reactively [37, 38]. Therefore, vaccination can prevent the emergence of novel drug-resistant phenotypes. Prediction models have suggested a 16% decrease in AMR-related typhoid fever after vaccination with TCV, equivalent to a potential reduction of 42 million cases and half a million deaths due to FQNS typhoid fever, and 21 million cases and 342 000 deaths from MDR typhoid fever over 10 years in countries eligible for Gavi support [39].

## CHALLENGES AND OPPORTUNITIES

### Tools for Typhoid Surveillance

Lack of disease burden data has long impaired typhoid control efforts [36, 40]. Several population-based studies have highlighted the heavy typhoid burden in many parts of Africa and Asia [41–44]. However, control efforts require local disease burden data, which are still missing in many parts of the world [36, 40, 45]. Monitoring disease burden to assess the impact of public health control measures requires diagnostic methods of high sensitivity and specificity. Unfortunately, blood culture, which is the principal diagnostic for typhoid fever, lacks sensitivity and is expensive, labor-intensive, and difficult to implement in settings where laboratory capacity is limited [36, 45, 46]. This highlights the pressing need for better diagnostic tools and, in particular, an accurate point-of-care test to support control efforts [47]. Finally, if typhoid elimination were an ambition, better methods to identify and treat asymptomatic carriers to interrupt *Salmonella* transmission chains are needed [48, 49]. Indeed, carriers are thought to maintain transmission in the community [48], require longer and more complex treatments [46], and complicate control (and elimination) efforts [49]. Of note, the impact of vaccination efforts will partly depend on their effectiveness in interrupting transmission by carriers, which is an important area for future research [50].

There have been recent advances in the development of surveillance tools for typhoid, which present opportunities to fill gaps in burden estimates. For example, serosurveillance for enteric fever is now possible, using dried blood spots from population-representative samples, which can be tested for antibodies to Hemolysin E, an antigen present in *S.* Typhi and *Salmonella* Paratyphi A [51]. Antibodies to Hemolysin E are elevated for many months after typhoid infection, with immunoglobulin (Ig)A typically decaying faster than IgG [52]. In a recent study, Aiemjoy et al [52, 53] measured longitudinal IgA and IgG antibody responses to Hemolysin E in confirmed enteric fever patients, and subsequently used the modeled antibody kinetic parameters to estimate age-specific seroincidence of typhoid in the general population based on cross-sectional population serology data, paving the way to approximate population-level incidence from serological data. There remain open questions about how these estimates should be interpreted in the absence of clinical evidence of severe disease, but this approach can be used rapidly in countries where no typhoid burden data exist, expanding the data available to local and global decision makers. Of note, Hemolysin E serology testing cannot discriminate between *S.* Typhi and *S.* Paratyphi A. As TCVs are being rolled out, serological (and diagnostic) tools able to differentiate *S.* Typhi from *S.* Paratyphi A are needed to establish respective disease burden, identify target populations, measure the impact of current vaccines, and guide development of future vaccines (potentially targeting multiple serovars).

Where no typhoid burden data exist, another promising approach is environmental surveillance [54–57]. Evidence of *S.* Typhi presence in sewage can be compelling evidence of current circulation of the pathogen in a community and hence the need to vaccinate. Whereas *S.* Typhi remains very difficult to culture from the environment, molecular detection approaches have been used [58], and these are currently being validated alongside blood culture-based surveillance in urban and rural populations in India, Malawi, Ghana, and Fiji [59].

### Vaccination and Water, Sanitation, and Hygiene to Control Typhoid

Although, historically, water, sanitation, and hygiene (WASH) have been instrumental in decreasing typhoid burden (and virtually eliminating it in some regions), typhoid remains a serious public health concern in many parts of the world [8, 18]. Vaccination has long been proposed as an excellent control tool [60], and the use of third-generation TCVs alongside WASH interventions seems particularly relevant in the current context of rising (multi-)drug resistance [26]. Recently, deployment of a TCV in children (6 months to 10 years) during an outbreak of XDR *S.* Typhi in Pakistan showed that, with an effectiveness of 95% against culture-confirmed *S.* Typhi, and 97% against XDR *S.* Typhi, vaccination was a powerful tool to curb disease spread in a densely populated region [61].

While WASH infrastructure improvements are often costly, technically difficult in densely populated urban areas, and only feasible in the long term [34, 62], vaccination may be quicker to implement, and modeling suggests that vaccination would be cost-effective in many endemic settings, in particular when disease burden is high [63, 64]. Cost-effectiveness model results were usually modulated by setting- and vaccine-related parameters such as typhoid incidence, contribution of carriers to transmission, probability of hospital admission, case-fatality ratio, type of vaccine rollout (routine vaccination with or without catch-up), efficacy of the vaccine used, duration of protection, and willingness to pay per-disability-adjusted life-year (DALY) averted [63, 65, 66]. Vaccination could be prioritized in the short term but should ideally be used in conjunction with longer term WASH infrastructure improvements to maximize the impact on typhoid burden [62, 63].

Of course, evidence of typhoid or enteric fever incidence in a community may be important input for decision makers planning water and sanitation infrastructure improvements and chlorination interventions [67], in addition to those in charge of vaccine introduction decisions. Improvements in infrastructure, water chlorination, and regulations on water and sewage treatment can lead to sustained and long-term reductions (and even elimination) of typhoid, as occurred in high-income settings in the 19th and early 20th centuries [68–70], and can also lead to reduction across multiple water-borne diseases such as cholera, in addition to typhoid [71]. Local and global leaders have an opportunity to address the long-standing need for water and sewage treatment in low-resource settings, by identifying funds—and creative funding mechanisms—that incentivize local governments to undertake water chlorination and infrastructure improvements. In an era of climate change and frequent high-intensity climate events, there is also a need for climate resilient, appropriate technology for rapidly growing urban centers in low-resource settings. There are potential implications of the COP27 Loss and Damage Funds in developing climate-resilient WASH infrastructure that can aid efforts for typhoid control.

In the case of typhoid, where no animal reservoir exists, the 2-pronged approach of (1) short-term disease reduction via vaccination and the (2) longer term reduction in the probability of environmental spread through water and sanitation improvement provide an opportunity to eliminate the disease in local contexts. A recent study in Dhaka, Bangladesh [41], a location with some of the highest reported typhoid incidence rates globally, showed that households that had a water filter and private toilet before vaccination, had a higher reduction in risk of typhoid incidence after TCV vaccination compared to houses without these improvements in water and sanitation [72]. Therefore, centralized improvements in community access to clean water and sanitation may act synergistically with TCV to reduce typhoid risk and incidence and could be tested before or after TCV introduction in contexts with a range of typhoid incidence rates.

### Further Considerations

Typhoid fever is often seen as a childhood disease, and this perception may lessen appetite for large-scale, all-ages vaccination efforts. In reality, although children below the age of 15 years bear the brunt of typhoid, a substantial disease burden is detectable in the over 15-year age group [41–44, 73, 74]. In countries or local areas where elimination is an ambition, vaccinating the adult population (ie, beyond 15 years of age) may provide additional opportunities to hasten a reduction in disease burden, an approach being piloted in some island settings such as Samoa [75]. Two TCVs (Typbar-TCV and Typhibev) are WHO prequalified and are recommended for programmatic use in endemic countries [32, 76]. Recent data reviewed by the WHO SAGE led to the conclusion that Typbar TCV is immunogenic not only in the age group 6 months to 45 years, but also among ages 46–65 years [30], presenting the opportunity to reduce disease and transmission in these subpopulations by vaccinating individuals above 45 years of age. However, more research and context-specific, real-world evidence will be instrumental to clarify (1) how expanding vaccination to older age groups impacts disease burden and (2) the cost-effectiveness of such a target age-group expansion.

Rapid urbanization worldwide adds another layer of complexity, because an increasing number of people live in typhoid-prone, poor, urban communities or slums where WASH infrastructure is deficient, and vaccine coverage is typically lower than in richer neighborhoods [62, 77]. Widespread rural-urban migrations result in pockets of susceptible urban populations, with low vaccine coverage (and lower health outcomes overall) [77, 78], and the potential for transmission of *S.* Typhi back to rural areas. This is particularly true in low- and middle-income countries where both urbanization and rural-urban migrations are common. It is ironic that this may create pockets of low vaccine coverage in areas where high coverage would be most desired, namely, low-income, high population density urban areas, which are particularly prone to infectious disease outbreaks. In these communities, the economic impact of outbreaks leads to disproportionately high levels of medical impoverishment among the most vulnerable due to higher out-of-pocket treatment expenditures as a proportion the household income [79]. In a world that is not constrained by vaccine supply, as may be the case with two WHO-prequalified TCVs and additional TCVs expected to be prequalified in the future [80, 81], improved access to vaccine in low-resource settings with support from Gavi, the Vaccine Alliance provides an opportunity to control this age-old scourge that continues to impact economically disadvantaged communities.

## CALL TO ACTION

The universal rise of AMR has unfortunately not bypassed *Salmonella*, which has been on the WHO list of antibiotic-resistant priority pathogens since 2017 [82], underscoring the urgency of a coordinated and comprehensive control plan. Controlling typhoid worldwide will require a multipronged approach including the availability of appropriate diagnostic and surveillance tools, the supply and large-scale delivery of prequalified TCVs, adequate strategies to reach high coverage, and the engagement of WASH funders.

Despite promising vaccine developments, urbanization and climate change have the potential to increase the global burden of typhoid fever [83]. Urbanization continues to outpace sanitation systems in many parts of the world, meaning that cities struggle to maintain adequate waste management and sewage removal systems. Meanwhile, as researchers have cautioned, climate change increases the risk for typhoid because of changes to storm patterns that increase the risk of flooding (associated with exposure to typhoid [84]) and drought, threatening safe drinking water and sewer systems.

New vaccines provide a lifeline during a time of failing antibiotics and rapid environmental change, but their rollout will have to be accompanied by other measures to move towards sustainable control of diseases that cause intestinal illnesses in low-resource countries [1]. Instead of an overreliance on any one intervention, wider control and elimination will be dependent on the provision of clean drinking water and waste-water systems, the implementation of WASH initiatives, an effective surveillance network, and the targeted provision of effective high-quality drugs and vaccines. Adopting this multi-intervention approach will crucially rely on community engagement [85], which is central to planning, implementing, and evaluating policy for disease prevention and control [86].
